# Factors contributing to the psychological well-being for Hong Kong Chinese children from low-income families: a qualitative study

**DOI:** 10.1186/s13033-016-0088-0

**Published:** 2016-09-08

**Authors:** Ka Yan Ho, William H. C. Li, Joyce Oi Kwan Chung, Katherine Ka Wai Lam, Sophia S. C. Chan, Wei Xia

**Affiliations:** School of Nursing, The University of Hong Kong, 4/F, William M. W. Mong Block, 21 Sassoon Road, Pokfulam, Hong Kong, China

**Keywords:** Income disparity, Poverty, Experience, Self-esteem, Quality of life

## Abstract

**Background:**

Despite compelling evidence demonstrating the negative impact of poverty and income disparity on children’s psychological well-being, there has been a lack of qualitative information which addresses its contributing factors. This study aimed to shed light on this area by comparing the experiences toward daily life between children living in low- and high-income families.

**Methods:**

A qualitative study using a phenomenological approach was conducted from May 2012 to January 2013. A random sample of 42 children aged 10–13, with 25 from low- and 17 from high-income families were asked to voluntarily response to a demographic sheet and undergo individual semi-structured interviews which lasted about 25–30 min. Content analysis was used to analyze the data. Approval for the study was obtained from the Institutional Review Board of the University of Hong Kong/Hospital Authority Hong Kong West Cluster (reference UW 12-237).

**Results:**

The findings of this study revealed that the living environment, physical health, social life and ability to function at school of children from low-income families are severely impaired.

**Conclusions:**

It fills a gap in the literature by showing how poverty and income disparity affect the daily lives of children from low-income families on different levels. Also, adopting a sedentary lifestyle and unhealthy eating habits are possible factors mediating the effects of poverty and income disparity on the psychological well-being of children from low-income families. It is vital for healthcare professionals going beyond their normal roles to give advice on healthy lifestyles and behaviors by building multidisciplinary partnerships with schools and the community. Additionally, healthcare professionals should also target on these two possible factors to develop and implement appropriate interventions for promoting the psychological well-being among children living in poverty.

*Trial registration* NCT02877719. 19 August 2016 retrospectively registered

## Background

It is well documented that poverty has a negative impact on children’s physical and psychological well-being [1–3]. There is also growing concern over the influence of income disparity on the psychological well-being of children, particularly for those living in low-income families [4, 5]. Research suggests that income disparity can actually aggravate the negative effects of poverty on children’s well-being, severely affecting their quality of life [4, 6, 7]. Nevertheless, despite the belief that income disparity is becoming a global problem, its relationship to the psychological well-being of children and their quality of life remains relatively underexplored.

Hong Kong has experienced an upward trend of poverty among its child population [8] and a serious income disparity with a Gini coefficient of 0.475 [9], the highest among the world’s developed economies. Results from a recent study by Ho and colleagues [5] examining the effects of poverty and income disparity on the psychological well-being of 1725 Hong Kong Chinese children reported that children from low-income families have statistically significant lower levels of self-esteem and quality of life, and higher levels of depressive symptoms than those from high-income families. Additionally, the results of this previous study also revealed that housing type and parent marital status were major factors contributing to children’s self-esteem, depressive symptoms, and quality of life [5].

The research of Ho and colleagues [5] provides some evidence that poverty and income disparity have a negative impact on the psychological well-being of children. However, their study is limited by a lack of qualitative information that might enable researchers to understand the problem more thoroughly. Specifically, they fail to address the reasons for children living in low-income families reporting lower levels of self-esteem and depression in the first place, subsequently leading to a diminished quality of life. Therefore, they pointed out the importance of collecting qualitative information to explore how poverty and income disparity affect children’s psychological well-being. Scientific evidence indicates the significance of examining the daily lives in reflecting and understanding ones’ psychological well-being [10]. In particular, it is recommended to review descriptions on the daily lives [11, 12], thus delineating the impact of poverty on children’s psychological well-being. More importantly, studying daily lives in relation to poverty and income disparity is essential prerequisites for the design of an effective and appropriate intervention that can help children living in low-income families to bolster their self-esteem, alleviate their depressive symptoms and improve their quality of life.

Given the foregoing issues, this follow-up study aimed to conduct qualitative interviews to enhance our understanding of how poverty and income disparity negatively affect the psychological well-being of Hong Kong Chinese children. It was anticipated that some underlying factors contributing to the psychological well-being of children from low-income families could be identified through comparing their daily lives with those from high-income families.

## Methods

### Design

A qualitative study using a phenomenological approach was conducted from May 2012 to January 2013. A random sample of 42 children aged 10–13, with 17 from families in the three highest monthly household income districts (high-income families) and 15 from the three lowest monthly household income districts (low-income families) were chosen from the previous study conducted by Ho and colleagues [5].

### Subjects

Hong Kong Chinese children who met the inclusion criteria were eligible for the interviews. The inclusion criteria were (1) able to speak and read Chinese, and (2) studying at grade five or six in elementary school. Also, they had to live in the same district where their elementary schools were located. This ensured that the informants were selected according to the study protocol, i.e., from families in the three highest and three lowest monthly household income districts. Those with identified behavioral or learning problems were excluded.

### Data collection procedures

Approval for the study was obtained from the Institutional Review Board of the University of Hong Kong/Hospital Authority Hong Kong West Cluster (reference UW 12-237). Written consent was then obtained from all parents after they were told the purpose of the study. They were given the option of allowing or refusing their child’s involvement in the study. The children were also invited to put their names on a special assent form and told that their participation was voluntary. The recruitment of informants was carried out in 12 elementary schools that were previously chosen from the three highest and three lowest monthly household income districts [5]. A name list of all eligible students was obtained from each school, and every student was assigned for a serial code. A computer programme would then use the serial codes for selection of students at random. All of the randomly selected students were asked to undergo individual semi-structured interviews conducted in a counseling room after school. All of the interviews were tape-recorded and lasted 25 to 30 min.

A semi-structured interview guide was developed. It covered seven areas of children’s life experience, including family background, living environment, usual diet, clothing, leisure activities, learning opportunities and personal feelings.

Two trained research nurses conducted all interviews. The informants were asked to respond to a broad question, “Can you share with me your everyday life other than going to school?” This was followed by more specific questions, such as, “How do you feel about your living environment?” During the interviews, the research nurses adopted different probing techniques to help elicit more comprehensive information.

### Analytic strategy

After the interviews, the recordings were immediately transcribed, verbatim, in Cantonese to capture nuances of expression unique to the dialect. Quotations relevant to the themes were then translated into English. In the coding process, two researchers who were not involved with data collection helped to analyze the narratives. They started with an extensive review of the transcripts, with the aim of searching for general constructs and themes. Special attention was paid to constructs and themes related to the major topics covered in the interview guide.

The technique of open coding was employed to code the transcriptions, followed by selective coding in which some categories were identified as core variables and used to code transcripts that were closely related to them. Regular meetings were held among the research team members to resolve any ambiguity or disagreement of coding.

## Results

### Characteristics of the informants

About 50 % of the informants were male, and the mean age was 11.6 years. Of the 42 informants, 25 came from low-income families and 17 came from high-income families. Most of their parents were married. Forty percent and 11.8 % of them having divorced or separated parents in low- and high-income families respectively. Of the informants from low-income families, a majority of their parents only attained lower secondary school (40.0 %) or even primary school or below (40.0 %), however most of the parents in high-income families received upper secondary school (35.3 %) or tertiary education (47.8 %). In terms of their housing types, whereas the interviewees from low-income families lived mainly in public rental housing (76.0 %), those from high-income families commonly lived in private housing as the owner occupiers (70.6 %).

Four themes were generated from the interviews: (1) living environment; (2) physical health; (3) social life; and (4) ability to function at school. Subthemes were further identified under each theme. Table 1 shows a presentation of themes, categories, and quotations for each category.

**Table 1:** A presentation of themes, categories, and quotations included in each category

		Children from low-income families	Children from high-income families
Theme	Category	Sample quotation	
Living environment	Living space for daily activities	*“I need to share a bedroom with my elder sister and I have to do my homework on the bed or in the corridor. My back is painful sometimes.”* *“I have to perform many of my daily activities, such as playing and studying, on a bed.”*	*“Although my home is not big, I do not have to share a bedroom or reading desk with my elder sister.”* *“I enjoy studying in my own bedroom. I also like my home environment. My mother always tidies the house and keeps it clean and safe for me to play and study.”*
Physical health	Physical activity	*“My parents ask me not to go out as it costs money. Therefore, I usually spend time watching television or sleeping in leisure time.”* *“I did not participate in any training course and I seldom go out, but I will exercise during physical education class.”*	*“During the weekend, they may take me to the countryside for outdoor activities.”* *“My parents always encourage me to participate in sport activities in leisure time and they told me that regular exercise is very important to me.”*
	Usual diet	*“I always have sausages, frozen meat and canned foods in dinner because they are cheap.”* *“My mother usually asks me to skip breakfast at the end of the month due to a lack of housekeeping money.”*	*“My parents seldom buy me canned or preserved food. They said that this food contains a lot of salt which is not good for my health.”* *“My mother usually makes me a sandwich for breakfast. Therefore, I always feel full in the early morning.”*
Social life	Material resources	*“I often wear the clothes of my elder brother. However, they don’t fit me. I have to roll up their sleeves before I put them on.”* *“My parents seldom buy me new toys. I often get old toys from my neighbors.”*	*“My parents often buy me clothes and sneakers from brand name stores.”* *“My mother just donated some of my clothes that are I wore for a few times to Po Leung Kuk (a NGO). She said my closet is crowded.”*
	Opportunity for children to take part in various kinds of activities	*“I am not able to attend school picnics or join any extracurricular activity. My mother tells me that all of these activities are too expensive.”* *“My parents said that transportation costs in Hong Kong are high. Therefore, I usually stay at home on weekends.”*	*“My parents always help me apply for different extracurricular activities. They said these activities can help broaden my horizons and enhance my competitiveness.”* *“I already went to Disneyland four times this year because I did well in my exams.”*
Ability to function at school	Stationery and educational resources	*“I always use the textbooks of my elder sister. Some of the content has not been updated.”* *“I do not have a computer at home. I have to go to public libraries to do online projects.”*	“*My parents often buy me new textbooks and school uniforms before the semester starts.”* *“My father bought a computer for me. Therefore, I have my own computer at home to complete online assignments from school.”*

### (T1) Living environment

#### Living space for daily activities

Many informants from low-income families considered their living environment unsatisfactory due to the inadequate space available for daily activities. Because most apartments in public housing estates have only one to two bedrooms, the majority said they had to share a room with their siblings and arranged their furniture and appliances in a compacted manner. Some informants living in subdivided flats even complained that they had to perform most of their daily activities on their bed or in a corridor with improper postures since there was not enough space in their home, thereby leading to shortsightedness and lower back pain.

Compared with the child informants from low-income families, those from high-income families were generally satisfied with their living environment. Because their private housing was relatively larger, most of them considered the living space available for daily activities to be adequate, and they said they had their own bedrooms. The majority also stated that their parents arranged the furniture and appliances in a tidy manner that reduced their risk of household injuries.

### (T2) Physical health

#### Physical activity

It was common for the informants from low-income families to adopt a sedentary lifestyle. To cut down on daily expenses, many of them reported that they were deprived of the chance to join training courses. Besides, the majority admitted to staying at home to watch television and play computer games during their leisure time. Some of them did not recognize the significance of regular physical exercise and considered themselves to be physically active when their only exercise was in physical education class.

In contrast, the child informants from high-income families were relatively active. Many of them said they were asked by their parents to join different sport activities after school and were brought outdoors for physical exercise on weekends. As many of their parents were better educated, quite a number of them were able to mention the advantages of regular physical activity and perceived physical activity to be very important.

#### Usual diet

Many informants from low-income families had unhealthy diets. To reduce daily expenses, they said it was common for their parents to buy canned foods and preserved vegetables that are high in sodium and cholesterol. Some informants also admitted to consuming expired food products as they were told by their parents not to waste food. Since their parents were less educated, the majority stated that they did not comprehend the disadvantages of an unhealthy diet, preferring to have meat and sugary foods for lunch and dinner.

When compared with the informants from low-income families, those from high-income families were likely to adopt healthier eating habits. The majority said their parents bought a variety of fresh foods and asked them to consume the proper amounts. Again, as many of their parents had received a tertiary education, quite a large number of the wealthier child informants acknowledged the importance of healthy eating and were able to articulate the consequences of an unhealthy diet.

### (T3) Social life

#### Material resources

Quite a number of the informants from low-income families could not afford material goods. Most of them mentioned that they had to wear the clothes of their elder siblings to save money. Some also said their parents seldom bought them toys and admitted to receiving second-hand toys from their neighbors. Because they lacked these material resources, many low-income informants stated that they would avoid making friends with children from high-income families, as they felt inferior.

In contrast, parents from high-income families were generally able to afford more material goods. Many of the child informants said they always wore brand-name clothing or fashionable shoes. Although some items were only worn a few times, quite a large number of informants reported that their parents might throw old clothes away when there was insufficient space for storage.

#### Opportunity for children to take part in various kinds of activities

The informants from low-income families were frequently deprived of the opportunity to take part in social activities. Due to limited financial resources, many of them said they were unable to participate in extra-curricular activities. Quite a large number of them also stated that they were asked by their parents to stay at home on weekends to cut down on unnecessary expenses. Although non-governmental organizations offered various kinds of activities for poor children, some were reluctant to join as they mentioned that they did not want to be stigmatized by their peers.

On the other hand, those from high-income families were given greater opportunity to join social activities. The majority reported having at least one extra-curricular activity, and said they could make new friends during the class. Many of these child informants also stated that their parents brought them to theme parks on weekends or even to trips during holidays.

### (T4) Ability to function at school

#### Stationery and educational resources

The educational resources enjoyed by informants from low-income families were generally insufficient. Many of them said they had to wear school uniforms and use textbooks from their elder siblings. Since there were discrepancies in content between different editions of textbooks, the majority said they needed to borrow updated versions and make photocopies. Some of them also stated that they did not want to wear their siblings’ uniforms because they did not fit or were too old, subjecting them to ridicule from their peers. Besides, many of the low-income informants reported lacking Internet access due to financial hardship, thereby finding it difficult to complete online assignments.

On the contrary, many informants from high-income families mentioned that their parents bought them new textbooks and school uniforms each academic year. Also, they had their own computers and Internet access at home, enabling them to obtain the most updated information for doing online assignments.

## Discussion

Although Ho and colleagues [5] investigated into the negative effects of poverty and income disparity on children’s psychological well-being, the main reasons behind remained unclear. To the best of our knowledge, this is the follow-up study is the first study that addresses the literature gap by demonstrating the possible underlying factors contributing to the psychological well-being of children from low-income families through comparing their daily lives with those from high-income families.

The results suggest that, in general, the daily lives of children from low-income families differ from those of children from high-income families in terms of their living environment, physical health, social lives and ability to function at school. Among them, living environment was the greatest concern reported by children from low-income families. This supports that housing type plays a significant role in children’s psychological outcomes [5]. Consistent with previous research [13, 14], informants from low-income families in our study generally considered their living environment to be unsatisfactory due to insufficient space for daily activities. Indeed, some evidence has suggested that a chaotic environment is a stressor contributing to distress [15, 16] by increasing the probability of family conflict [17] and household injuries [18]. Since this is uncontrollable, children living in such environments may perceive themselves as not having any ability to change things, thereby feeling depressed [19].

Another possible explanation for poor psychological outcomes in underprivileged children is limited access to material resources and social activities. Many informants from low-income families in our study reported being unable to afford material goods and were also discouraged from participating in various kinds of social activities to cut down on unnecessary expenses. Indeed, this lack of access may have a greater than expected negative impact on children’s psychological well-being [20]. According to Festinger [21], humans are motivated to make comparisons with other society members in terms of their financial condition. Those considering themselves in a better position obtain a sense of superiority, which helps build up their self-esteem; whereas exclusion from services may create a sense of inadequacy, thereby lowering a child’s self-esteem [22, 23]. This state of mind was observed in the semi-structured interviews, as those having difficulty accessing material resources and social services reported avoiding making friends with children from high-income families and feeling inferior due to their financial situation.

This study also provides further insight into how a parent’s marital status may affect a child’s psychological outcome [5]. As indicated by the demographic data, 40 % of informants from low-income families compared with only 11.8 % from high-income families had divorced or separated parents. According to Thomas and Sawhill [24], family structure can directly affect household income, with single-parent families reporting less income. It is therefore understandable that children from these families may be more deprived of material resources and social services compared with those from married-parent families, resulting in feelings of inferiority and susceptibility to the psychological effects of poverty and income disparity.

One of the interesting findings was that many children from low-income families had unhealthy eating habits and also adopted a sedentary lifestyle. As observed in the interviews, it was common for these children to consume high-sugar foods and spend their leisure time watching television. Indeed, it has been well-documented that physical inactivity is associated with depressive symptoms [25] and low self-esteem [26]. Although the reason is unknown, engaging in regular physical exercise has been recognized as an effective way to reduce stress [27] by stimulating the secretion of endorphins, which leads to feelings of happiness [28]. Additionally, there is some evidence that healthy eating is linked to higher self-esteem as it can reduce the risk of obesity, thereby improving self-image [29]. Given the above issues, adopting a sedentary lifestyle and unhealthy eating habits are possible factors mediating the effects of poverty and income disparity on the psychological well-being of children from low-income families.

Another finding warranting special attention is that quite a large number of informants from low-income families complained about their difficulty accessing educational resources. Indeed, scholars have suggested that such disadvantages may deprive children of opportunities to move up the social ladder, rendering them much more susceptible to intergenerational poverty [30]. This provides further justification for supporting a greater allocation of resources by the government to children from low-income families, thereby helping them to break the cycle of poverty.

### Limitations

This study has several limitations. First, the interviews were only conducted with children. Because of their immature cognitive development, they might have been unable to provide a detailed explanation of how poverty and income disparity affected their psychological well-being. We recommend that parents and social workers be included in future studies to obtain a better understanding of their effects on children. Another limitation is that all of the informants were from families in the three highest and three lowest monthly household income districts. The daily lives of children from the middle range thus remain unknown. Future studies could consider recruiting them as informants to show how poverty and income disparity impact on the daily lives and psychological well-being of children from a wider range of socioeconomic backgrounds.

### Implications for research and clinical practice

An important implication of this research is that it adds to the literature by showing that a sedentary lifestyle and unhealthy eating habits are possible factors mediating the effects of poverty and income disparity on the psychological outcomes of children from low-income families. Future research can integrate them as new variables for evaluating this segment of the population.

Understanding how poverty and income disparity affect the daily lives of children from low-income families should also be a prerequisite for designing an appropriate intervention to promote their psychological well-being. As physical inactivity and unhealthy eating habits are two possible reasons for poor psychological outcomes, developing a protocol advocating regular physical exercise and healthy eating is of paramount importance.

More importantly, nurses should make themselves aware of the effect of poverty and income disparity on children’s psychological health, forming multi-disciplinary partnerships with different stakeholders to promote children’s psychological well-being.

One more important implication relates to highlight the significance in improving the material circumstances of low-income families especially in terms of living environment, social and educational resources. Children’s rich descriptions on these areas guide the development of suitable measures to address their particular concerns. Considerations should also be given to the study results when planning services and policies in the long run.

## Conclusions

This study fills a gap in the literature by showing how poverty and income disparity affect the daily lives of children from low-income families on different levels. More importantly, adopting a sedentary lifestyle and unhealthy eating habits are possible factors mediating the effects of poverty and income disparity on the psychological well-being of children from low-income families. It is vital for healthcare professionals going beyond their normal roles to give advice on healthy lifestyles and behaviors by building multidisciplinary partnerships with schools and the community. Additionally, healthcare professionals should also target on these two possible factors to develop and implement appropriate interventions for promoting the psychological well-being among children living in poverty.
